# Effect of short‐term endurance training on venous compliance in the calf and forearm differs between continuous and interval exercise in humans

**DOI:** 10.14814/phy2.14211

**Published:** 2019-09-11

**Authors:** Anna Oue, Michiko Saito, Yasuhiro Iimura

**Affiliations:** ^1^ Faculty of Food and Nutritional Sciences Toyo University Gunma Japan; ^2^ Graduate School of Food and Nutritional Sciences Toyo University Gunma Japan

**Keywords:** exercise training, venous capacitance, venous distensibility, venous outflow

## Abstract

We examined whether the effect of short‐term endurance exercise training on venous compliance in the calf and forearm differed between continuous and interval workloads. Young healthy volunteers (10 women and 16 men) were randomly assigned to continuous (C‐TRA; n = 8) and interval (I‐TRA; n = 9) exercise training groups, and a control group (n = 9). Subjects in the C‐TRA group performed a continuous cycling exercise at 60% of heart rate reserve (HRR), and subjects in the I‐TRA group performed a cycling exercise consisting of alternating 2‐min intervals at 40% HRR and 80% HRR. Training programs were performed for 40 min/day, 3 days/week for 8 weeks. Before and after training, limb volume in the calf and forearm was measured with subjects in the supine position by venous occlusion plethysmography using a venous collecting cuff placed around the thigh and upper arm. Cuff pressure was held at 60 mmHg for 8 min and then decreased to 0 mmHg at a rate of 1 mmHg/s. Venous compliance was calculated as the numerical derivative of the cuff pressure–limb volume curve. Calf venous compliance was increased after I‐TRA, but not C‐TRA. Forearm venous compliance was unchanged after C‐TRA or I‐TRA. These results suggest that the adaptation of venous compliance in response to endurance training for 8 week may occur in interval but not continuous exercise bouts and may be specific to the exercising limb.

## Introduction

Habitual exercise is a well‐established intervention for reducing risk factors for cardiovascular disease (Mora et al., 2007) and improving arterial vascular function and structure (Green, 2009). Traditionally, moderate‐intensity continuous training has been recommended (Pescatello et al., 2004; Haskell et al., 2007), and has increased endothelial function (Tinken et al., 2008; Birk et al., 2012), decreased arterial stiffness (Kakiyama et al., 2005), and improved arterial dilator capacity as surrogate measure for structural remodeling (Tinken et al., 2008). Recently, some studies have shown that high‐intensity aerobic interval training is superior to continuous training for reducing arterial stiffness as measured by carotid‐femoral pulse wave velocity (PWV) (Ciolac et al., 2010; Guimarães et al., 2010) and improved the flow‐mediated vasodilation (FMD) of brachial artery (Tjønna et al., 2008).

Compared with arteries, veins have high compliance and contain approximately 60‐70% of the total blood volume at rest (Greenfield and Patterson, 1956; Morris, Abrecht, and Leverett, 1974). The response of the venous system to various physiological stresses may play an important role in the control of circulation (Rothe, 1983). In addition to the adaptation of arterial vessels due to regular exercise, some cross‐sectional studies have reported that calf venous compliance was greater in trained subjects who engaged in habitual endurance exercise for at least 2 years compared with sedentary subjects (Monahan, Dinenno, Seals, and Halliwill, 2001; Hernandez and Franke, 2004), indicating that chronic endurance exercise could also enhance venous compliance. In addition, some longitudinal studies have shown that increased calf venous compliance in the exercising limb was also observed after continuous exercise training (C‐TRA) for 6‐8 weeks (Iida et al., 2011; Scholten, Hopman, Lotgering, and Spaanderman, 2015), although forearm venous compliance in the non‐exercising limb was unchanged after C‐TRA for 6 weeks (Iida et al., 2011). These previous studies suggest that the adaptation of venous compliance in response to C‐TRA may occur specifically in the exercising limb; however, the effect of interval exercise training on venous compliance in exercising and non‐exercising limbs is not well understood. The larger gradients of shear stress between high and low intensities during interval exercise are thought to cause larger responses at the cellular and molecular level, so that the greater endothelial function with increased bioavailability of nitric oxide (NO) during the interval than C‐TRA could be observed (Wisløff et al., 2007). Indeed, some studies reported the greater improvements in arterial stiffness after high‐intensity aerobic interval training rather than moderate‐intensity continuous training (Ciolac et al., 2010; Guimarães et al., 2010). Like this, because adaptation of endothelial function with training might differ between interval and continuous exercise, we hypothesized that the effect of short‐term endurance training on venous compliance in exercising limb and non‐exercising limb will also differ between interval and continuous exercise.

Thus, to test our hypothesis, we investigated changes in venous compliance in the calf (exercising limb) and forearm (non‐exercising limb) before and after 8 weeks of C‐TRA or I‐TRA.

## Methods

### Ethical approval

This study was approved by the Human Ethics Committee of Toyo University (approval no. 2015‐K‐02) and was conducted in accordance with the Declaration of Helsinki except for registration in a database. All subjects were given detailed explanations about the purpose, procedures, and risks of the study, and all provided written and oral informed consent.

### Subjects

A total of 26 young healthy volunteers (10 women and 16 men) participated in the study. We excluded subjects who had overt chronic diseases as assessed by their medical history and a physical examination. Subjects were randomly assigned to the C‐TRA (n = 8, 3 women and 5 men), the I‐TRA (n = 9, 4 women and 5 men), and a control group (CONT; n = 9, 3 women and 6 men).

### Study design

Subjects in the C‐TRA and I‐TRA groups performed cycling exercise training for 8 weeks, whereas subjects in the CONT group maintained their usual lifestyle without special exercise for 8 weeks. Physical working capacity at 170 beats/min (PWC_170_) and venous vascular properties (venous compliance, venous capacitance, and venous outflow) were assessed for all subjects before (Pre) and after (Post) the training period.

### Incremental cycling test

All subjects performed a submaximal incremental cycling test before and after the training period in order to determine the training workload and assess the PWC_170_ such as physical capacity. Subjects were asked to pedal on a cycle ergometer (Ergomedic 828E, Monark Exercise AB, Vansbro, Sweden) at a constant frequency of 60 rpm for 3 min at four different exercise intensities. Workloads at 1st, 2nd, 3rd and 4th stages corresponded to approximately 100~110 bpm, 120~130 bpm, 140~150 bpm, and 160~170 bpm of heart rate (HR) during exercise, respectively. Throughout the protocol, HR was measured by a heart rate monitor (Polar F11, Polar, Kempele, Finland). Workloads corresponding to 60% HR reserve (HRR) for the C‐TRA group and 40% HRR and 80% HRR for the I‐TRA group were calculated. HRR was calculated as the difference between peak and resting HR, multiplied by the intensity of exercise and then added to resting HR, according to the Karvonen method for calculating HR (Karvonen, Kentala, and Mustala, 1957). In addition, PWC_170_ was estimated by extrapolating the line of best fit to the four submaximal HR‐workload data points.

### Assessment of venous vascular properties

Subjects rested in the supine position for at least 20 min before data acquisition. Each subject's left leg and left forearm were placed slightly above the heart level and supported at the ankle and wrist, respectively. To measure change in volume in the calf and forearm, a venous collecting cuff was wrapped around the left thigh and left upper arm, and a mercury strain gauge was placed on the sites of maximal thickness of the calf and forearm. Next, to place the strain gauge at the same position before and after the training period, we recorded the location of the strain gauge before the training period, and then matched the position after the training period. The collecting cuff was inflated to 60 mmHg for 8 min, after which the cuff pressure was linearly reduced manually from 60 mmHg to 0 mmHg at a rate of 1 mmHg/s in all trials according to a previously described cuff deflation protocol (Halliwill, Minson, and Joyner, 1999). Limb volume during cuff inflation and deflation was measured noninvasively by venous occlusion plethysmography (EC6, D. E. Hokanson, Bellevue, WA). All limb volume data were recorded on a personal computer using an analog‐to‐digital converter (15BX, Dacs Electronics Co., Ltd., Okayama, Japan). A rapid increase in limb volume was evoked within 3–4 min of elevating cuff pressure to 60 mmHg, representing the maximal blood volume stored in the veins at the given pressure, followed by a slower, fairly linear volume increase caused by net filtration into the extravascular space. We calculated this filtration‐dependent increase using a model developed by Skoog et al. (2015), and corrected the limb volume during inflation and deflation of cuff pressure.

Using the corrected limb volume curve, we assessed venous vascular properties as described below. The relationship between cuff pressure and change in volume in the calf and forearm (i.e., the pressure–volume curve) was generated from the data points between 10 mmHg and 60 mmHg during the cuff deflation protocol. To avoid any a priori assumptions regarding the pressure (P)–limb volume (V) curve and obtain a physiologic venous compliance curve, venous compliance was calculated as the numerical derivative of each pair of pressure–limb volume data points using the following equation (Freeman, Lirofonis, Farquhar, and Risk, 2002).

$$\text{Venous}\;{\text{compliance}}_{\mathrm{Pi}}=\frac{V_{i}-V_{i-5}}{P_{i}-P_{i-5}},\;\text{where}\;15\leq i\leq 60$$

Venous capacitance was evaluated as the change in limb volume from before the cuff inflation to 8 min of cuff inflation at 60 mmHg. Venous outflow was calculated from the rate of change in limb volume for 1 min during cuff deflation from 60 to 0 mmHg.

In pilot study, we investigated the coefficient of variation (CV) of plethysmography approach in nine healthy subjects. Within the same day (separated 2~3 h), CV was 3.3% (0.5–10.9%) in calf and 5.9% (0.4–13.8%) in forearm. In addition, between days (separated 1wk), CV was 3.6% (0.6–16.0%) in calf and 5.3% (3.0–14.4%) in forearm. For biological variables, a CV of <10 % is considered good and <20 % is acceptable (Scott, Randolph, and Leier, 1989).

### Exercise training

C‐TRA consisted of continuous cycling exercise at 60% HRR for 40 min on an ergometer, 3 days per week for 8 weeks. I‐TRA consisted of alternating 2‐min intervals of cycling exercise at 40% HRR and 80% HRR for 40 min, 3 days per week for 8 weeks, resulting in a mean workload of 60% HRR. The incremental cycling test was readministered at 4 weeks from the start of training, and training loads were adjusted to maintain a training intensity equivalent to 60% HRR for the C‐TRA group and 40% HRR and 80% HRR for the I‐TRA group. Throughout training, HR was monitored by a heart rate monitor (Polar F11, Polar). In addition, each subject was asked to rate perceived exertion (RPE) on a Borg scale of 6‐20 (Borg, 1970) every 4 min during exercise in both C‐TRA and I‐TRA.

### Measurements

Systolic (SBP) and diastolic (DBP) arterial blood pressure and HR at rest were measured with subjects in the supine position before the measurement of change in limb volume during the cuff deflation protocol. SBP and DBP were measured from the left brachial artery by brachial auscultation using a sphygmomanometer (KM‐380, Kenzmedico, Saitama, Japan). HR was monitored by a heart rate monitor (Polar F11, Polar).

### Data analysis and statistics

Data are expressed as mean ± standard error (SE). In C‐TRA, HR during exercise was averaged every 4 min. In I‐TRA, HR during exercise was averaged every 2 min at each low and high intensities, respectively, and then the averaged HR every 4 min during exercise involving in both workloads was calculated. Time courses of HR in C‐TRA and I‐TRA during cycling exercise were averaged in all training trials for 8 weeks, respectively. In addition, time course of RPE during exercise was also averaged for 8 weeks. SBP, DBP, and HR at rest and PWC_170_, net fluid filtration flow, venous capacitance, and venous outflow were compared between Pre and Post within the C‐TRA, I‐TRA, and CONT groups using paired *t*‐tests. To compare changes in limb volume and venous compliance with cuff pressure between Pre and Post within the C‐TRA, I‐TRA, and CONT groups, two‐way analysis of variance (ANOVA) with repeated measures was applied to the venous volume and venous compliance obtained with cuff pressure of 10‐60 mmHg under each condition (Pre and Post), using cuff pressure and condition as fixed factors. If main effects and/or interaction of conditions were detected, a post hoc analysis using a paired *t*‐test was performed every 5 mmHg. To compare changes in venous compliance at 20 mmHg of cuff pressure from Pre to Post among groups (C‐TRA, I‐TRA, and CONT), one‐way ANOVA was used, and if main effect was detected, post hoc analysis using a Tukey test was performed. To compare time courses of HR and RPE every 4 min during exercise between C‐TRA and I‐TRA, two‐way ANOVA with repeated measures was applied, using time and condition as fixed factors. If main and/or interaction effects were detected, a post hoc analysis using Mann‐Whitney's *U* test was performed every 4 min. Statistical analysis was performed using SPSS software version 19 (IBM Corp., Armonk, NY). A value of *P* < 0.05 was considered statistically significant.

## Results

### Subject characteristics at rest in Pre and Post

Table 1 shows subject characteristics in Pre and Post. PWC_170_ in Post was increased to a similar degree in the C‐TRA and I‐TRA groups (*P* < 0.05 for both). Resting HR decreased in the I‐TRA group (*P* < 0.05) but not in the C‐TRA group. There was no difference in weight or resting BP between Pre and Post in the C‐TRA and I‐TRA groups. In the CONT group, all parameters were similar between Pre and Post.

**Table 1:** Subject characteristics at rest in Pre and Post

	C‐TRA		I‐TRA		CONT	
	Pre	Post	Pre	Post	Pre	Post
Age (years)	21.5 ± 0.8	‐	20.1 ± 0.2	‐	22.1 ± 0.3	‐
Height (cm)	169.4 ± 3.6	‐	167.0 ± 3.1	‐	164.0 ± 2.3	‐
Weight (kg)	63.7 ± 2.6	63.5 ± 2.7	60.0 ± 2.8	60.5 ± 2.9	57.2 ± 1.9	57.1 ± 1.8
Systolic blood pressure (mmHg)	120 ± 3	116 ± 3	118 ± 3	122 ± 4	120 ± 4	118 ± 4
Diastolic blood pressure (mmHg)	71 ± 2	69 ± 1	69 ± 2	68 ± 1	74 ± 2	74 ± 2
Heart rate (beats/min)	73 ± 4	66 ± 3	69 ± 3	63 ± 2*	79 ± 3	74 ± 3
PWC_170_ (Watt/kg)	2.02 ± 0.22	2.76 ± 0.17*	2.35 ± 0.19	3.02 ± 0.17*	2.24 ± 0.14	2.27 ± 0.12

Values are presented as the mean ± standard error. PWC_170_, Physical working capacity at 170 beats/min.

* *P* < 0.05 for Pre vs. Post.

### HR and RPE during cycling exercise in C‐TRA and I‐TRA

**Figure 1:**
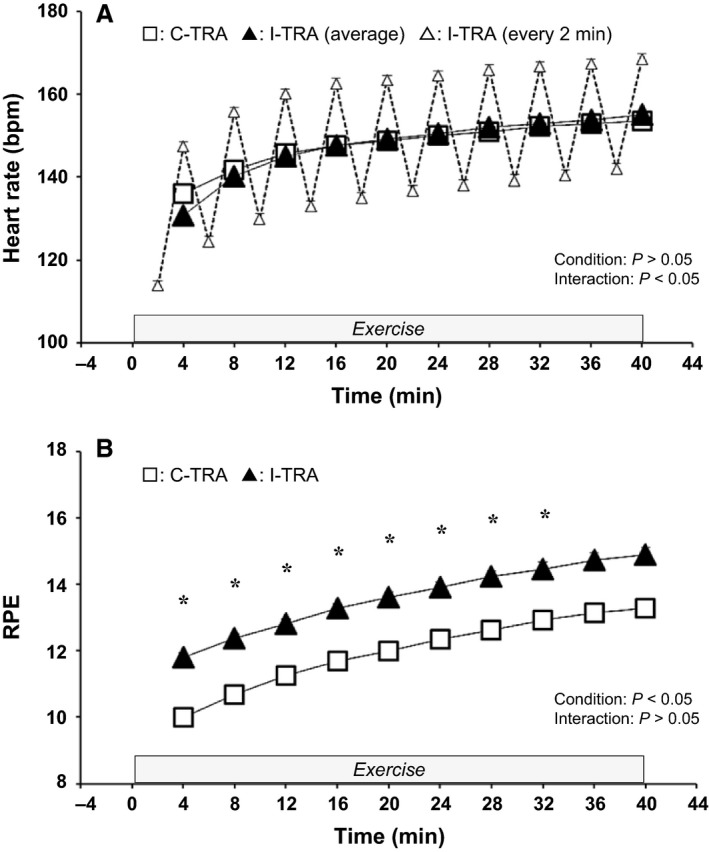
Time courses of heart rate and rate perceived exertion (RPE) during cycling exercise in C‐TRA or I‐TRA. Each data was averaged in all training trial for 8 weeks. □: HR every 4 min in C‐TRA. △: HR every 2 min at low and high workloads in I‐TRA. ▲: averaged HR in low and high workloads in I‐TRA. Values are presented as the mean ± standard error. *P < 0.05 for C‐TRA vs. I‐TRA

Figure 1 shows time courses of HR and RPE during cycling exercise in C‐TRA and I‐TRA for 8 weeks. HR every 2 min during exercise in I‐TRA group varied according to exercise intensities. However, the averaged HR every 4 min in I‐TRA was almost similar to HR every 4 min in C‐TRA (Fig. 1A). ANOVA also yielded a significant interaction effect for mean HR between C‐TRA and I‐TRA (*P* < 0.05), although a post hoc test showed no significant difference. RPE was increased gradually during exercise in both C‐TRA and I‐TRA, and the time course of RPE was greater in I‐TRA than C‐TRA (Fig. 1B). For RPE between C‐TRA and I‐TRA, ANOVA yielded a significant condition effect (*P* < 0.05) and a post hoc test showed a significant difference at exercise time of 4‐32 min (*P* < 0.05).

### Effect of continuous exercise training on venous vascular properties

**Figure 2:**
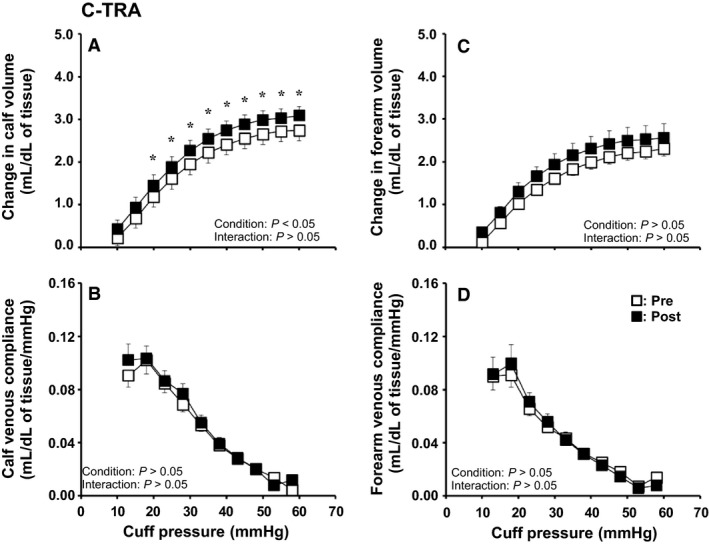
Cuff pressure–limb volume curves and cuff pressure–venous compliance relationships in the calf (A, B) and forearm (C, D) before (Pre) and after (Post) continuous exercise training (C‐TRA) for 8 weeks. Values are presented as the mean ± standard error. *P < 0.05 for Pre vs. Post

Figure 2 shows the effect of C‐TRA on limb volume and venous compliance in the calf and forearm. The cuff pressure‒calf volume curve shifted upward after C‐TRA (Fig. 2A), but the cuff pressure‒calf venous compliance curve was unchanged (Fig. 2B). For mean calf volume between Pre and Post, ANOVA yielded a significant condition effect (*P* < 0.05) and a post hoc test showed a significant difference at cuff pressures in the range of 20–60 mmHg (*P* < 0.05). In addition, calf venous capacitance was significantly higher in Post than in Pre (*P* < 0.05), although calf venous outflow was similar between Pre and Post (Table 2). In contrast, all venous vascular properties of the forearm were unchanged after training (Fig. 2C and 2D, and Table 2).

**Table 2:** Venous capacitance and venous outflow in Pre and Post

	C‐TRA		I‐TRA		CONT	
	Pre	Post	Pre	Post	Pre	Post
Calf						
Venous capacitance (mL/dL of tissue)	2.74 ± 0.25	3.08 ± 0.22*	3.40 ± 0.31	3.85 ± 0.34*	3.16 ± 0.35	3.40 ± 0.43
Venous outflow (mL/dL of tissue/min)	3.08 ± 0.17	3.41 ± 0.23	3.76 ± 0.32	4.19 ± 0.32	3.29 ± 0.30	3.89 ± 0.41
Forearm						
Venous capacitance (mL/dL of tissue)	2.31 ± 0.17	2.58 ± 0.32	2.45 ± 0.17	2.85 ± 0.28	3.47 ± 0.39	3.54 ± 0.46
Venous outflow (mL/dL of tissue/min)	2.62 ± 0.21	2.73 ± 0.30	2.81 ± 0.17	2.85 ± 0.19	3.31 ± 0.31	3.57 ± 0.37

Values are presented as the mean ± standard error.

* *P* < 0.05 for Pre vs. Post.

### Effect of interval exercise training on venous vascular properties

**Figure 3:**
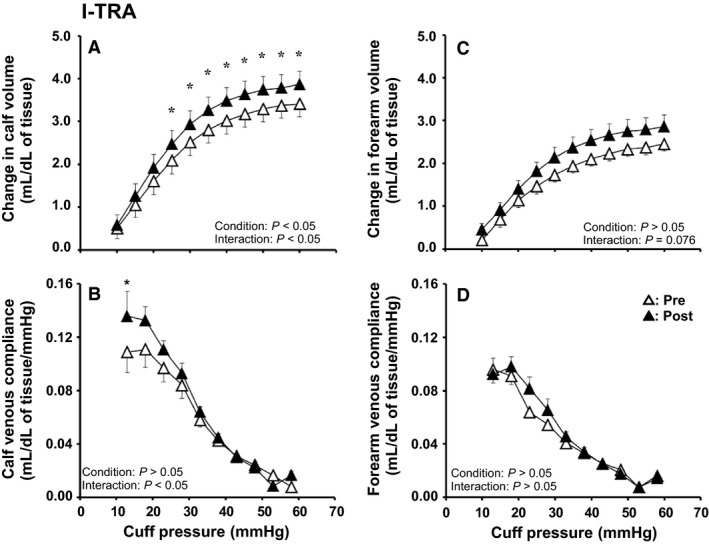
Cuff pressure–limb volume curves and cuff pressure–venous compliance relationships in the calf (A, B) and forearm (C, D) before (Pre) and after (Post) interval exercise training (I‐TRA) for 8 weeks. Values are presented as the mean ± standard error. *P < 0.05 for Pre vs. Post

Figure 3 shows the effect of I‐TRA on limb volume and venous compliance in the calf and forearm. The cuff pressure‒calf volume and cuff pressure‒calf venous compliance curves shifted upward after I‐TRA (Fig. 3A and 3B). For mean calf volume between Pre and Post, ANOVA yielded significant condition of main effect (*P* < 0.05) and interaction effects (*P* < 0.05) and a post hoc test showed a significant difference at cuff pressures in the range of 25‐60 mmHg (*P* < 0.05). ANOVA also yielded a significant interaction effect for mean calf venous compliance between Pre and Post (*P* < 0.05), and a post hoc test showed a significant difference at a cuff pressure of 15 mmHg (*P* < 0.05). In addition, ANOVA also showed that the change in calf venous compliance at 20 mmHg of cuff pressure from Pre to Post had significant difference among groups (*P* < 0.05), and post hoc test revealed that the change in calf venous compliance from Pre to Post was significantly greater in I‐TRA than CONT (*P* < 0.05), but there was no significant difference between C‐TRA and I‐TRA or CONT (I‐TRA: 0.031 ± 0.009 mL/dL of tissue/mmHg, C‐TRA: 0.006 ± 0.009 mL/dL of tissue/mmHg, CONT: –0.025 ± 0.019 mL/dL of tissue/mmHg). Calf venous capacitance was significantly increased after I‐TRA (*P* < 0.05), although calf venous outflow was similar between Pre and Post (Table 2). In the forearm, the cuff pressure‒volume curve shifted upward slightly after I‐TRA. For mean forearm volume between Pre and Post, ANOVA tended to yield interaction effects (*P* = 0.076) (Fig. 3C). However, venous compliance, venous capacity, and venous outflow in the forearm were unchanged after interval training (Fig. 3D, and Table 2).

**Figure 4:**
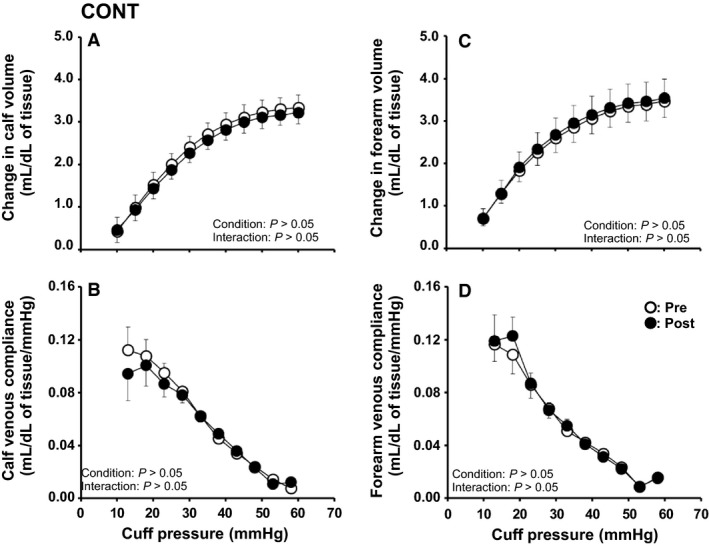
Cuff pressure–limb volume curves and cuff pressure–venous compliance relationships in the calf (A, B) and forearm (C, D) before (Pre) and after (Post) usual lifestyle (CONT) for 8 weeks. Values are presented as the mean ± standard error

In the CONT group, there were no significant differences between Pre and Post for all venous vascular properties of the calf and forearm (Fig. 4 and Table 2).

## Discussion

New findings from the present study are as follows. First, calf venous compliance was increased after I‐TRA but not after C‐TRA. Second, calf venous capacitance was enhanced after both I‐TRA and C‐TRA, although there was no difference in calf venous return between before and after training. Third, forearm venous compliance was not altered after C‐TRA or I‐TRA, but forearm volume tended to increase after I‐TRA but not after C‐TRA. These results indicate that the adaptation of venous compliance in response to 8 weeks of endurance training in healthy young subjects may be caused by the interval exercise, but not continuous exercise, and may occur specifically in the exercising limb.

### Effect of short‐term endurance cycling training on calf venous adaptation in the exercising limb

Calf venous compliance increased after I‐TRA, but not after C‐TRA (Fig. 1B and 2B). Cross‐sectional studies have shown that habitual endurance exercise training can lead to increased calf venous compliance (Monahan, Dinenno, Seals, and Halliwill, 2001; Hernandez and Franke, 2004), although the time frame for such adaptation is not well understood. Some studies have reported that endurance training for 6‐8 weeks increased calf venous compliance in older subjects (Iida et al., 2011) and formerly preeclamptic women (Scholten, Hopman, Lotgering, and Spaanderman, 2015), who already had lower venous compliance before exercise training. To our knowledge, this present study is the first to demonstrate that interval endurance exercise training for 8 weeks can enhance calf venous compliance in healthy young subjects, who would likely have relatively higher calf venous compliance before training. However, given that calf venous compliance was unchanged after 8 weeks of continuous endurance exercise training, further investigation is needed to elucidate the intensity, frequency, and duration of exercise training required to effectively improve venous compliance.

It is possible that the increased calf venous compliance observed with I‐TRA, but not C‐TRA, is due to differences in blood flow response and shear stress during these types of exercise. Previous studies have indicated that greater improvements in arterial stiffness (i.e. carotid‐femoral PWV) are obtained with interval rather than C‐TRA (Guimarães et al., 2010). In addition, Wisløff et al. (2007) suggested that the greater endothelial function observed during interval training compared with C‐TRA might be due to increased bioavailability of NO, since the larger gradients of shear stress created between high and low intensities during interval exercise might trigger larger responses at the cellular and molecular level. Although findings regarding arterial adaptation cannot be directly applied to veins because of differences in vascular structure, variations in blood flow response and shear stress between interval exercise and continuous exercise may have contributed in part to the increased calf venous compliance observed after I‐TRA but not C‐TRA in the present study.

In addition, the muscle pump is an important mechanism for assisting venous return and providing perfusion of skeletal muscle during rhythmic exercise (Laughlin 1987). For example, it is reported that the muscle pump might decrease venous pressure by 55–65 mmHg in the human legs during rhythmic exercise (Folkow, Haglund, Jodal, and Lundgren, 1971). In addition, mean arterial blood pressure during exercise is elevated with the increase in workload. Considering to these, as exercise intensity is higher, the higher perfusion and greater venous return could be caused by muscle activity and muscle pump. I‐TRA program consists of exercise bouts at low and high intensities. That is, the greater increase in venous return could be obtained by the muscle pump during high workload in I‐TRA, which might cause greater improvement of endothelium function in vein via shear stress. Like this, since the more effective changes in muscle activity, muscle pump, and venous return might occur during interval rather than continuous exercise, the significant influence of exercise training on venous compliance might occur in I‐TRA but not C‐TRA in our study.

Contrary to the change in venous compliance, calf volume was increased after both I‐TRA and C‐TRA (Fig. 1A and 2A). Since the increases in the cross‐sectional area (CSA) of the abdominal inferior vena cava (Miyachi, Okutsu, Nakahara, and Saitoh, 1999) and femoral vein (Miyachi et al., 2001) were observed after endurance training for 8–12 weeks, venous expansion is likely to be caused by not only I‐TRA but also C‐TRA. In contrast, an animal study revealed that continuous endurance training for 9 weeks induced not only increased capillary numbers but also increased capillary luminal diameter, and this adaptive response of capillary diameter to endurance training was dependent on training intensity (Kano et al., 1997). In the present study, because calf volume measured by venous occlusion plethysmography reflects the vascular responses of all arteries, veins, and capillaries in the whole limb, we cannot exclude the possibility that the increased capillary volume due to changes in number and/or diameter after both interval and continuous training for 8 weeks could result in increased calf volume and venous capacitance in both I‐TRA and C‐TRA.

### Physiological mechanism for increased venous compliance with short‐term endurance exercise training

Although the physiological mechanisms, by which short‐term interval aerobic exercise training causes increased venous compliance, are less clear, the contribution of structural and/or functional factors can be hypothesized. Such as structural adaptation, vascular remodeling might be considered. A previous study showed that mechanical distension due to increased blood flow during exercise may increase arterial compliance by stretching and modifying the cross‐linking of collagen fibers (Brüel, Ortoft, and Oxlund, 1998). In addition, Hanna et al. (2014) reported that 10 weeks of endurance exercise training in young rats improved arterial stiffness and myogenic function even though characteristics of the arterial wall, such as the collagen‐to‐elastin ratio, were not altered. Considering these reports, it may be inferred that 8 weeks of short‐term endurance training in our study could induce venous vascular remodeling, such as changes in collagen cross‐linking.

As with functional adaptation, changes in the sympathetic nervous system and endothelial function should also be considered. First, increased compliance with training could result from modulation of the sympathetic adrenergic tone of smooth muscle cells. However, many previous studies have shown no change in resting muscle sympathetic nerve activity after 4‐16 weeks of longitudinal aerobic exercise training (Svedenhag, Wallin, Sundlöf, and Henriksson, 1984; Sheldahl, Ebert, Cox, and Tristani, 1994; Ray, 1999; Cooke et al., 2002; Ray and Carter, 2010). In addition, it has been reported that calf venous compliance in response to adrenergic blockade did not increase at rest (Sielatycki, Shamimi‐Noori, Pfeiffer, and Monahan, 2011). Considering these reports, it could be speculated that sympathetic adrenergic mechanisms did not directly contribute to the increased calf venous compliance observed with exercise training in the present study. A second possibility is enhancement of endothelial release of NO. In human and animal studies, short‐term exercise training has been shown to enhance endothelium‐dependent dilation responses in both arterioles and conduit vessels (Wang, Wolin, and Hintze, 1993; Koller, Huang, Sun, and Kaley, 1995; Green et al., 2017), increase NO production, and endothelial nitric oxide synthase (eNOS) gene expression in coronary arterioles (Sessa et al., 1994), and augment eNOS protein levels in skeletal muscle arterioles and the aorta (Sun, Huang, Koller, and Kaley, 1994; Delp and Laughlin, 1997). Although these findings were reported in arterial vessels, it is possible that vascular remodeling (i.e. changes in collagen cross‐linking) and adaptation of endothelial function to short‐term exercise training might also occur in veins, which could have contributed in part to the increased calf venous compliance seen with I‐TRA in the present study.

### Effect of short‐term endurance cycling training on forearm venous adaptation of the non‐exercising limb

Consistent with previous findings (Iida et al., 2011), forearm venous compliance was unchanged after I‐TRA and C‐TRA (Fig. 1D and 2D), suggesting that the adaptation of venous compliance in response to short‐term endurance training might be specific to the exercising limb. However, the change in forearm volume in I‐TRA tended to be higher in Post than in Pre (P = 0.076) (Fig. 2C). In addition to our results, a previous study also reported that one‐leg high‐intensity (80% of maximal oxygen uptake) endurance cycling exercise training for 6 weeks caused increased CSA in the femoral vein of both the exercising and non‐exercising legs (Miyachi et al., 2001). From these findings, we speculate that some adaptation of venous function and/or venodilator capacity without changes in venous compliance might also occur in response to short‐term exercise training not only in the exercising limb but also in the non‐exercising limb.

Regarding findings of adaptation of the brachial artery to cycling exercise training for 8 weeks, FMD increased from Pre at 2 weeks after high‐intensity exercise training (80% HRmax or 80% HRR), and then returned to Pre level at 8 weeks after exercise (Tinken et al., 2008; Birk et al., 2012), although vasodilator capacity increased gradually from Pre across the training intervention period (Tinken et al., 2008). Additionally, the increased FMD in the non‐exercising upper arm with training might be mediated, at least partly, by shear stress (Birk et al., 2012). Given that the increased venous function with exercise training could also be modulated systemically by shear stress, the degree of venous adaptation in the non‐exercising limb might be greater in the skin rather than in muscle because blood flow in the superficial (skin) veins increased, but blood flow in the deep (muscle) veins remains unchanged in the non‐exercising upper arm during prolonged cycling exercise with increased blood flow in the brachial artery (Ooue et al., 2008). Thus, speculatively, venous vascular adaptation to exercise training might occur systemically, but the unchanged venous adaptation in muscle area might mask the increased venous adaptation in the skin area in the non‐exercising limb because of competitive blood flow between both sites; changes in venous volume and/or compliance with exercise training might thus be smaller in the non‐exercising limb than the exercising limb. Furthermore, considering the enhanced FMD after high‐intensity exercise training (Tinken et al., 2008; Birk et al., 2012), and the tendency toward increased forearm volume after I‐TRA, but not C‐TRA, in our study, venous adaptation might be achieved after high‐intensity exercise training.

### Significance of increased venous compliance with exercise training

Our findings may have important physiological and clinical significance. Aging and physiological inactivity might cause stiffness of the veins (Hernandez and Franke, 2004; Monahan, Dinenno, Seals, and Halliwill, 2001; Olsen & Lanne, 1998; Tsutsui et al., 2002; Young, Stillabower, DiSabatino, and Farquhar, 2006), which could be a factor in the pathogenesis of hypertension (Olsen & Lanne, 1998; Safar and London, 1987). Besides artery, venous compliance improvement could be also a significant marker for overall cardiovascular health. Indeed, verifying the results from previous cross‐sectional studies (Monahan, Dinenno, Seals, and Halliwill, 2001; Hernandez and Franke, 2004) and longitudinal studies (Hernandez and Franke, 2005; Iida et al., 2011; Scholten, Hopman, Lotgering, and Spaanderman, 2015), our findings also indicate that habitual endurance exercise training is likely to be a beneficial intervention for improving venous compliance, which could contribute to preventing lifestyle diseases that may be related to elevated venous stiffness, such as hypertension (Safar and London, 1987; Olsen & Lanne, 1998). On the other hand, high venous compliance and capacitance could induce greater amount of blood pooling in the legs at a high rate in an upright posture, likely causing greater orthostatic stress to arterial blood pressure regulation leading to orthostatic intolerance. Thus, it is necessary to further investigate how increased venous compliance with exercise training influences blood pressure regulation.

### Limitations

This study has some limitations. First, we did not confirm whether there was equal metabolic energy requirement during exercise sessions between interval and continuous workloads. Because the energy efficiency of cycling may be lower during interval training than during continuous training, metabolic demands could differ between I‐TRA and C‐TRA. However, this possibility is likely to be nearly negligible because the degree of increased PWC_170_, which is an index of physical capacity, was similar between I‐TRA and C‐TRA. Second, we did not consider the menstrual cycle in experiments with women. In addition, previous studies report that calf venous compliance in men at low cuff pressure was higher than that in women (Monahan and Ray, 2004; Meendering et al., 2005; Lindenberger & Länne, 2007), and our study also shows that forearm venous compliance at a cuff pressure of 20 mmHg was greater in men than women (men: 0.111 ± 0.008 mL/dL of tissue/mmHg; women: 0.076 ± 0.004 mL/dL of tissue/mmHg; *P* < 0.05). However, the percent change in venous compliance from pretraining to posttraining with both interval and C‐TRA was similar between men and women. Thus, we believe that sex differences did not influence the changes in venous compliance in response to exercise training in our study, even though the possibility that inclusion of both sexes might mask slight differences cannot be excluded. Third, although we discussed the possible physiological mechanisms for improvement of venous compliance with exercise training, this speculation was based on findings related to arterial adaptation and has never been directly verified in venous vessels. In order to clarify these points, future studies should specifically investigate the adaptation of venous compliance in response to exercise training.

## Conclusion

This study demonstrated that I‐TRA for 8 weeks, but not C‐TRA, increased calf venous compliance in young adults while forearm venous compliance remained unchanged. These findings suggest that the adaptation of venous compliance in response to short‐term endurance training could be induced by not moderate continuous exercise but interval exercise at low and high intensities, and might be specific to the exercising limb.

## Conflict of Interest

The authors have no financial conflict of interest to declare.
